# The outcomes of a low-cost, non-valved glaucoma drainage device using mitomycin-C: 1-year results

**DOI:** 10.1007/s00417-023-06019-y

**Published:** 2023-03-04

**Authors:** Mo’mena Ahmad A. Awad-Allah, Amr Saleh Mousa, Doaa Maamoun Ashour

**Affiliations:** Ophthalmology Department, Faculty of Medicine, Ain Shams University, Cairo, Egypt

**Keywords:** Aurolab Aqueous Drainage Implant (AADI), Ripcord, Mitomycin-C, Low-cost implant

## Abstract

**Purpose:**

To evaluate the indications, outcomes, and complications of the usage of Aurolab Aqueous Drainage Implant (AADI) using mitomycin-C**.**

**Methods:**

A retrospective case series of patients who underwent AADI placement using mitomycin-C between April 2018 and June 2020 at Ain Shams University Hospitals, Cairo, Egypt. The data was extracted from the records of the patients with a minimum of 1 year of follow-up. Complete success was defined as IOP ≥ 5 mmHg and ≤ 21 mmHg or reduction of IOP by ≥ 20% from baseline without antiglaucoma medications (AGMs). Qualified success was defined as reaching the same IOP range with the aid of AGM.

**Results:**

A total of 50 eyes of 48 patients were included. Neovascular glaucoma represented the commonest indication (13 patients, 26%). The mean preoperative IOP was 34.0 ± 7.1 mmHg, with a median number of AGM of 3 (mean ± SD = 2.84 ± 1), while the mean IOP after 12 months was 14.3 ± 4 with a median number of AGM of 0. (mean ± SD = 0.52 ± 0.89) (*p* < 0.001). Complete success was achieved in 33 patients (66%). Qualified success was achieved in 14 patients (28%). Thirteen eyes (26%) had variable postoperative complications; none of them required explantation of the device or affected the visual acuity (except one patient).

**Conclusion:**

AADI with using mitomycin-C and ripcord during the surgery is an effective and relatively safe method of control of IOP in refractory and advanced cases of glaucoma, with an overall success rate of 94%.


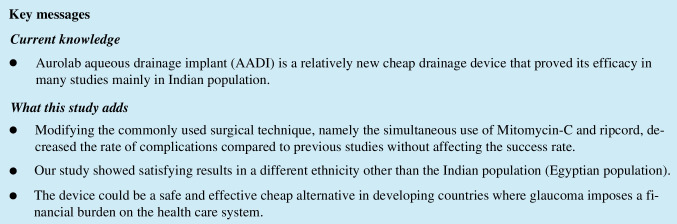


## Introduction

Glaucoma drainage devices (GDDs) are currently main players in the management of glaucoma, either as a primary or secondary surgical intervention [1, 2]. The most used GDDs worldwide are Ahmed glaucoma valve (AGV, New World Medical, Rancho Cucamonga, CA) and Baerveldt glaucoma implant (BGI, Johnson & Johnson Vision, New Brunswick, NJ). Many studies have reported the safety and the efficacy of both devices and/or compared their results to conventional glaucoma surgery [1, 3, 4]. The BGI has shown a lower failure rate and fewer postoperative antiglaucoma medication requirement [5]. According to the surface area of its end plate, BGI had originally three models: 500 mm^2^, 350 mm^2^, and 250 mm^2^. Long-term follow-up has shown better results for the 350-mm^2^ BGI than the 500-mm^2^ implant for intraocular pressure (IOP) control, and currently, it is the commonest model in practice [6].

Despite the well-validated role of GDD, especially in refractory glaucoma, the relatively high cost of GDD limits their use in developing countries [7]. Treatment costs, either medical or surgical, have a major contribution to the economic burden of glaucoma, which may eventually affect the outcome of the patients [8]. In the developing countries, owing to the limited resources and the lack of awareness, management plans may vary from those made for the same cases in the developed countries [9, 10].

In 2013, a low-cost none valved GDD was introduced to the market in India: the Aurolab Aqueous Drainage Implant (AADI, Aurolab, Madurai, India). The design of this device is based on the BGI 350 mm^2^ but with a much lower cost (about 70 dollars per device). Few studies, mainly from India, reported that this device is safe and effective in different types of glaucoma [7, 11–13]. In this study, we report the 1-year safety and efficacy of the AADI using mitomycin-C and insertion of a ripcord in the tube as modifications of the originally described technique among Egyptian glaucoma patients.

### Methods

This is a real-world retrospective non-comparative interventional case series. All patients who underwent AADI placement between April 2018 and June 2020 at the Department of Ophthalmology, Ain Shams University Hospitals, Cairo, Egypt, were included. The study protocol was approved by the Research Ethics Committee of the Faculty of Medicine, Ain Shams University. The study adhered to the research ethics stated by the declaration of Helsinki. All patients or their guardians (if less than 18 years old) signed written informed consent before their shunt device surgery.

The medical records and the surgical details for all patients who underwent AADI placement during the designated period were extracted. All patients who completed at least a 1-year follow-up period were included. Demographic data including the patients’ age, gender, residency, and family history of glaucoma were extracted. The data regarding type and duration of glaucoma, number of antiglaucoma medications (AGMs), and history of any previous eye surgery were collected. The data of the baseline ophthalmological assessment, including best-corrected visual acuity (BCVA), anterior segment assessment with the slit lamp, and the last recorded preoperative IOP using the Goldmann applanation tonometer (Keeler ltd., Windsor, UK) or Perkins tonometer Mk2 (Haag-Striet UK Ltd., UK) in pediatric cases were extracted. The surgical notes were revised, and any intraoperative complication was recorded. The follow-up records for all visits up to 1 year or more were revised. Data regarding BCVA, IOP, the number of antiglaucoma medications (AGMs), the need to remove the prolene suture, and any postoperative complications were extracted and analyzed. Records with missing preoperative data, operative details, or IOP at any visit were excluded from the study while records with missing data regarding BCVA or AGM were included in the study with exclusion from the statistical analysis of these variables.

### Surgical technique

The surgical technique described by Pathak Ray and Rao was used with very few differences, mainly, the use of mitomycin-C and ripcord [7]. After checking the patency, prolene 3-0 suture (Surgiopro™, Medtronic, USA) was inserted inside the tube for initial control of aqueous flow through the AADI, to be released when needed according to IOP (acting as a ripcord), and its free end was fashioned into a loop that was fixed to the sclera with a 9-0 nylon (GMS, Egypt) mattress stitch, for easy retrieval later. The tube was ligated with a 6-0 Vicryl suture (AssuCryl®, Assut sutures, Switzerland). After conjunctival dissection in the planned quadrant, mitomycin-C (Biochem Pharma, India) with a concentration of 0.3 to 0.4 mg/ml (according to the severity of conjunctival affection from previous surgeries) was applied for 2 min, followed by a thorough wash. Devices were placed in the supero-temporal quadrant in most cases. The infero-nasal implantation was chosen in eyes with severe conjunctival scarring, extensive peripheral anterior synechiae in the supero-temporal angle of the anterior chamber, or silicone-filled eyes. Anterior chamber placement of the tube was done in all cases except for three cases that needed pars plana insertion. In those cases, the tube was further shortened and a slightly slanting forward track was created 4 mm from the limbus. In all the patients, the implant was inserted beneath the recti muscles except in one eye with microspherophakia with an axial length of 20.7 mm and the lateral rectus had a congenital anomaly and was posteriorly inserted (10 mm from the limbus) so the decision was to place the implant over the muscles in this eye.

Postoperative topical steroids topical (Pred Forte eye drops, prednisolone acetate 1%, Allergan, Irvine, CA) and antibiotics (Tymer eye drops, gatifloxacin 0.3%, Jamjoom Pharma, KSA) were prescribed for 3 weeks. IOP was measured on the first day postoperatively, and AGMs were adjusted according to the IOP. Most of the patients were kept on their preoperative antiglaucoma regimen with the addition of oral acetazolamide if needed.

### Outcome measures

The primary outcome was IOP reduction. Complete success was defined as IOP ≥ 5 mmHg and ≤ 21 mmHg or reduction of IOP by ≥ 20% from baseline without any AGM. Qualified success was defined as reaching the same IOP range with the aid of AGM.  Failure of the surgery was defined as loss of vision (no light perception), the need for a second glaucoma surgery, or tube explantation during the follow-up period [7].

A hypertensive phase (HTP) was defined as a rise of IOP > 21 mm Hg, with a high tense cystic bleb around the plate. This may start after 10 days of surgery and persist up to 6 months requiring AGM for control of IOP. The subsequent reduction in bleb height, with step-down of AGM, or discontinuation, was defined as resolving or resolved HTP [14].

Data from the postoperative visits at 1 week, 2 months, 6 months, and 1 year were extracted and analyzed.

### Statistical analysis

The data was statistically analyzed using the Statistical Package for Social Science (SPSS 20). The descriptive statistics were presented as mean, standard deviation (± SD), median and interquartile range, or as frequency and percentage for non-numerical data. A *P* value less than 0.05 was considered statistically significant. The Kaplan-Meier graph was plotted to show the survival of the AADI over 1 year in terms of total success (complete and qualified) and complete success.

## Results

In this study, a total of 50 eyes of 48 patients (29 males and 19 females) were included. The mean age was 42.1 ± 20.2 years, with a range from 1.5 to 66 years. A family history of glaucoma was present in 6 patients, two of them with congenital glaucoma.

The surgery was done on 29 right eyes (58%) and 21 left eyes. The mean glaucoma duration since the first diagnosis was 4.9 ± 6.2 years, with a range from 3 months up to 28 years. The different etiologies of glaucoma in the included sample are shown in Table 1. Neovascular glaucoma represented the commonest indication for AADI placement in the included sample (13 patients, 26%).

**Table 1:** Etiologies of glaucoma in the included sample

Etiologies of glaucoma	Frequency	Percent
Congenital	10	20%
Silicon induced	12	24%
Neovascular	13	26%
Secondary to a complicated keratoplasty	6	12%
POAG	3	6%
Others: angle recession, 2ry to microspherophakia in a case of Weil Marchesani, uveitic, angel resection, unidentified cause in a case of Steven Johnson syndrome	6	12%
Total	50	100%

*POAG* primary open angle glaucoma

Thirty-nine eyes (88%) had previous intraocular surgery. Vitrectomy was the most common prior surgery (16 patients, 32%). About a quarter of the patients (13 patients, 26%) had a previous one or more glaucoma surgery. Six patients (12%) had a previous penetrating keratoplasty, and 4 patients had phacoemulsification with the intraocular lens implantation surgery.

**Fig. 1:**
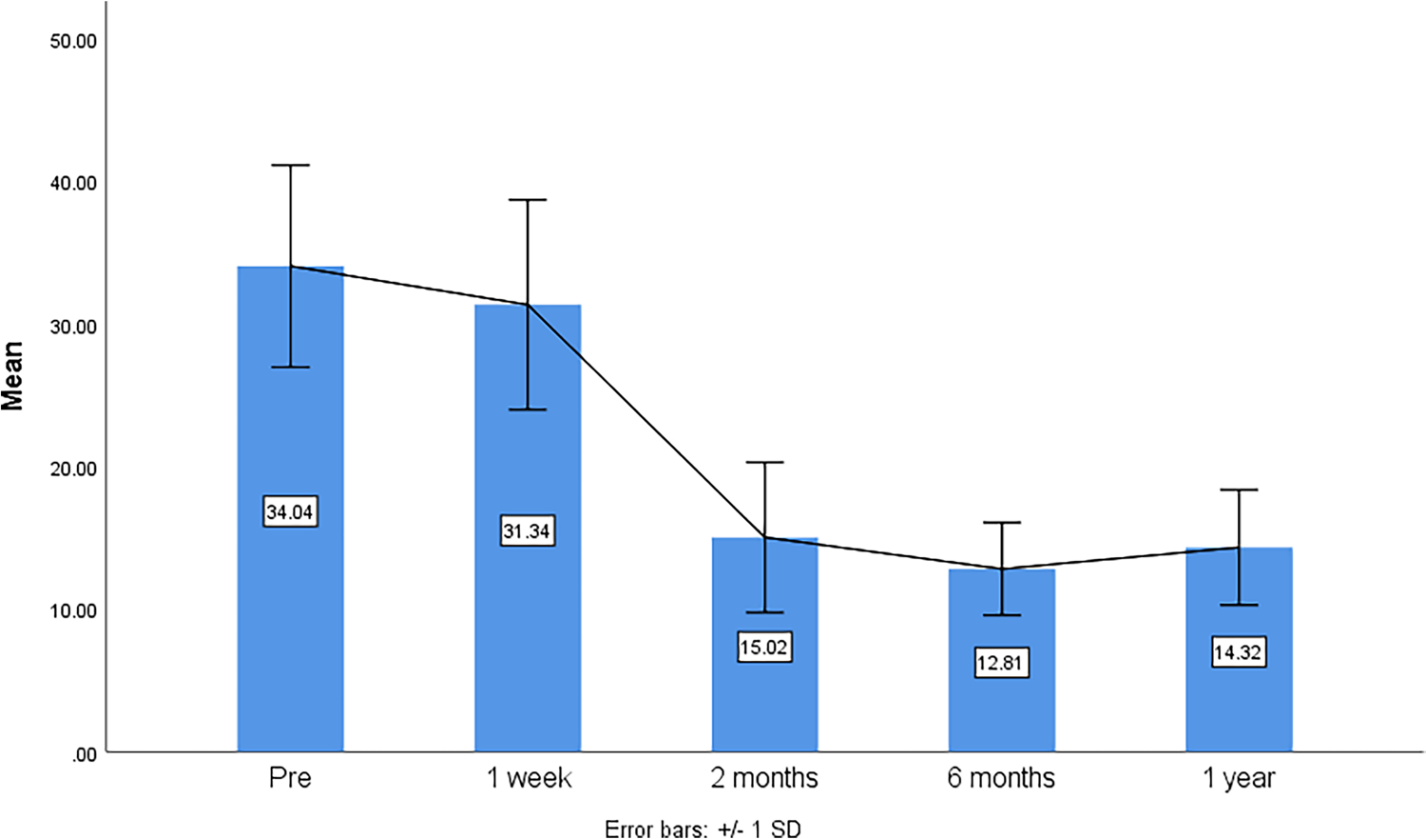
The mean IOP (intraocular pressure) preoperative and at 1 week, 2 months, 6 months, and 1 year

The mean follow-up duration was 12.9 ± 1 months. A highly statistically significant reduction of the IOP was achieved (*p* < 0.001). The mean preoperative IOP was 34.0 ± 7.1 mmHg, with a median number of AGM of 3 (mean ± SD = 2.84 ± 1), while the mean IOP after 12 months was 14.3 ± 4 with a median number of AGM of 0 (mean ± SD = 0.52 ± 0.89) (Table 2) The mean IOP reduction was 18.3 mmHg. The pattern of IOP changes and reduction in the follow-up visits (1 week, 2 months, 6 months, and 1 year) is shown in Fig. 1.

**Table 2:** Preoperative and postoperative IOP analysis

	IOP pre	IOP final visit
Mean	34.04	14.27
Median	32.00	14.00
SD	7.089	4.015
Minimum	24	8
Maximum	48	32

*IOP* intraocular pressure, *SD* standard deviation

Best corrected visual acuity was recorded from 49 eyes after exclusion of a preverbal child. Using one-way ANOVA test, no significant changes in the BCVA was observed (*p* value = 0.85) between the preoperative BCVA 1.39 ± 0.58 (49 eyes), BCVA at 1 week 1.48 ± 53 (49 eyes), at 2 months 1.34 ± 57 (37 eyes), at 6 months 1.38 ± 58 (32 eyes), and the postoperative BCVA 1.38 ± 0.59 (49 eyes).

**Fig. 2:**
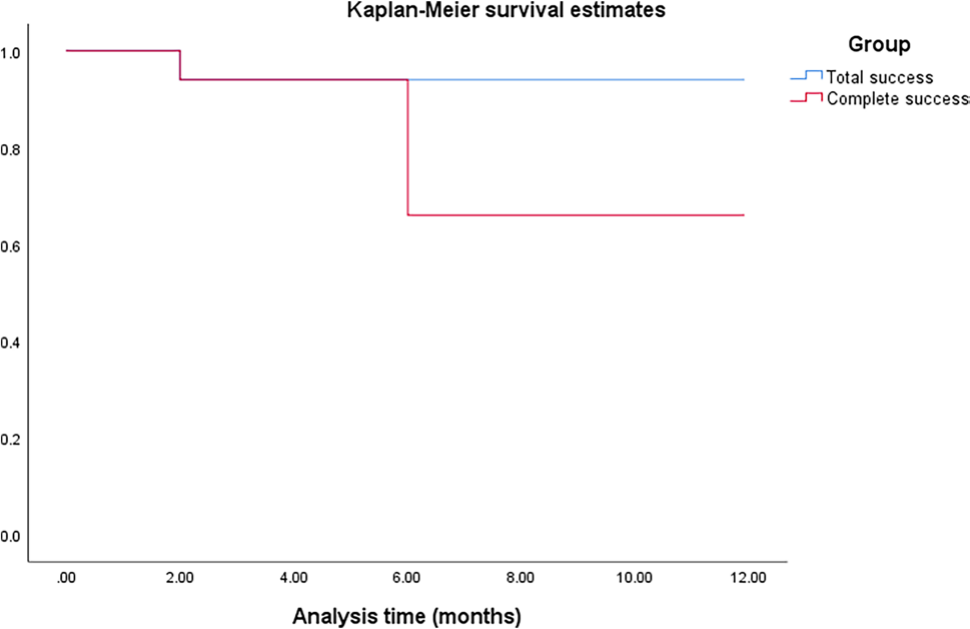
Kaplan–Meier survival graph showing the survival of the AADI over 1 year in terms of total success (complete and qualified) and complete success

Complete success, as previously defined, was achieved in 33 patients (66%). One of those 33 patients had a severe early encapsulation after 4 months and underwent excision of this capsule after which a good control of the IOP was achieved for up to 14 months. Accordingly, this patient was included in the complete success group. Three patients had a transient period of low IOP (one of them showed manifestations of hypotony) due to the early release of the Vicryl suture ligating the tube during the first 2 weeks postoperative. All of them achieved spontaneous resolution within 2 weeks with a final IOP at 1 year within the range of complete success. Qualified success was achieved in 14 patients (28%). The total success rate was 94% (95% confidence interval (CI) at 1 year of 84.8–98.3%). Failure was encountered in 3 eyes, two patients needed diode laser cyclophotocoagulation in the first year because of uncontrolled IOP, and a uveitic patient developed extensive peripheral anterior synechiae due to uncontrolled iridocyclitis. The tube opening was partially occluded with a final IOP of 24 mmHg on quadruple therapy, and he was the only patient to have a significant drop of vision (dropped from counting fingers at 25 cm to light perception). Kaplan-Meier survival curve (Fig. 2) shows the rates of total (complete and qualified success) and complete success over 1 year from the implantation of AADI.

There was a decrease in number of AGM starting from the second month follow-up compared to the preoperative number. There was some missing data for 6 eyes at the 6-month follow-up; accordingly, the statistical analysis included 44 eyes only at this point. Analysis of number of AGM is shown in Table 3.

**Table 3:** Analysis of number of antiglaucoma medications used per visit

	Mean ± SD	Median	IQR
Preoperative (50 eyes)	2.84 ± 1.00	3	2–3
1 week (50 eyes)	2.90 ± 0.97	3	3–3
2 months (50 eyes)	0.40 ± 0.78	0	0–1
6 months (44 eyes)	0.36 ± 0.81	0	0–0
1 year (50 eyes)	0.52 ± 0.89	0	0–1

*SD* standard deviation, *IQR* interquartile range

A transient hypertensive phase, as defined before, occurred in 7 patients (14%) between the sixth and the twelfth week. All of them were medically controlled and resolved within 1 to 3 weeks.

No considerable intraoperative complication occurred in any of the patients. Thirty-seven eyes (74%) had an uneventful postoperative period. While in 13 eyes (26%) variable postoperative complications occurred as described in Table 4 as early (occurred within the first month postoperative) and late complications, with the timing of occurrence in each patient. Seven patients (14%) underwent another surgical intervention or procedure; two underwent cyclophotocoagulation, four underwent conjunctival re-suturing, and one underwent dissection of valve encapsulation.

**Table 4:** Recorded postoperative complications (early and late) with the timing of occurrence

Complications	Frequency	Percent	Timing of occurrence
*Early complication (within 1 month):*			
Conjunctival opening without tube exposure	1	2.0	2nd week
Early opening of the device (without hypotony)	2	4.0	1st week and 2nd week
Early hypotony and cataract progression	1	2.0	2nd week
Uncontrolled uveitis with uncontrolled IOP (extensive anterior synechiae)	1	2.0	1st week
*Late complications:*			
Cataract progression	1	2.0	9th month
Uncontrolled IOP	2	4.0	4th month
Severe encapsulation	1	2.0	4th month
Silicon egression	1	2.0	2nd month
Tube exposure	3	6.0	3rd, 6th, and 9th month
* No complications*	37	74.0	

*IOP* intraocular pressure

The removal of the prolene suture ripcord from the tube was done in only 15 eyes (30%) during the follow-up period. The earliest removal was done 3 months after surgery.

## Discussion

In Egypt, the only available drainage device was the Ahmed valve till the AADI was introduced to the Egyptian market in 2017 at approximately one-third of the cost of the Ahmed valve. To our knowledge, this is the largest report of the outcome of this device among the Egyptian population.

Neovascular glaucoma was the commonest indication for AADI placement among the studied cohort (26%), followed by silicone-induced glaucoma (24%) and congenital glaucoma (20%). Previously, GDDs were found to be safe and effective in the management of neovascular and silicone-induced glaucoma [15, 16]. Having secondary glaucoma, either neovascular or silicone-filled as the commonest indication might be related to the pattern of referral in Ain Shams Glaucoma Clinic as previously reported by Ashour et al. [17].

There was an overall statistically significant reduction in IOP (mean IOP reduction of 18.3 mmHg at 12 months postoperative). These results are consistent with the results of Pathak Ray and Rao as well as the results reported by Pandav et al. [7, 12]. In both studies, there was a highly statistically significant reduction in IOP at 12 months of follow-up. Also, the results are consistent with those obtained by Puthuran et al. who had the mean IOP decreased to 15.10 ± 6.7 mmHg at 1 year postoperative [18]. In the current study, an overall reduction in the number of medications was observed. Thirty-four eyes (68% of the eyes) maintained good IOP control with no medications. The total success rate was 94%, which is similar to the success rate obtained by Ray (92.6%) [7]. Another IOP lowering procedure was needed in only 2 patients.

The AADI showed a high safety profile with few postoperative complications. Most of the complications were either self-limited or required minimal intervention with no effect on the final IOP control at 1 year. Tube exposure occurred in only three patients. One of them had an early severe reaction and melting of the scleral graft used, and it was replaced by another one with no further complications. In this case, there was no accompanying reaction in the AC or the capsule over the plate, so this graft reaction was attributed to poor storage of the used scleral graft. The other two cases of tube exposure were in a 5-year-old child and an adult with a mental disability, who were reported by their caregivers to be constantly rubbing their eyes. To the best of our knowledge, the only report of AADI outcome in the Egyptian population included only congenital glaucoma children by Rateb et al. [19]. In their study, severe anterior chamber reaction non-responsive to medical treatment was reported in 37% of the patients. We did not encounter any case of severe reaction except in one patient who was already an uncontrolled uveitic patient. This difference in results may be attributed to the very young age of the patients (less than 28 months of age) included in that study. We had also a lower rate of complications compared to the study conducted by Ray who had an overall complications rate at 1 year of 40.7% [7]. This difference may be attributed to the differences in the used surgical technique such as the use of ripcord that decreases the incidence of hypotony and the application of patch graft that might decrease the rate of exposure.

In our study, the transient hypertensive phase occurred in only 14% of patients and subsided with medical treatment within 1 to 3 weeks. This percentage is lower than that found by Senthil et al. (occurred in 19% of his pediatric group of patients) and Puthuran et al. (32% of their patients) [20, 21]. This lower incidence might be attributed to the difference in the surgical technique used in our study as mitomycin-C was applied to all patients. The adjuvant use of mitomycin-C with AGV has been associated with a lower incidence of postoperative hypertensive phase [22]. In addition to the effective role of antimetabolites in glaucoma surgeries among the different African populations [23, 24], to the best of our knowledge, no previous studies compared AADI with and without the adjuvant use of antimetabolites. However, based on the cumulative experience in the Egyptian glaucoma patients, antimetabolites were used in the whole included sample.

The incidence of the transient hypertensive phase is claimed to be lower in non-valved implants such as Molteno and Baerveldt compared to the Ahmed valve [18, 25]. Nouri-Mahdavi and Caprioli reported a high incidence of hypertensive phase following implantation of Ahmed valve (56%) [25]. As the design of AADI is inspired by the design of the 360-mm surface area model of Baerveldt, a low incidence of hypertensive phase was expected. The hypertensive phase could be a crucial contributor to the poor outcome of GDD surgeries as the high pressure could cause further damage to the already affected nerve fibers. It also correlates to the long-term IOP control as it is an indicator for the encapsulation surrounding the device plate.

Since we used mitomycin-C, we added a prolene ripcord for better IOP control. The analysis of the outcome of this study shows that this technique might be the most appropriate (at least in the Egyptian population) for decreasing the incidence of postoperative hypotony. In our study, hypotony occurred in only 3 eyes due to the early release of the Vicryl suture ligating the tube within the first 2 weeks postoperative, and it subsided without further surgical intervention within a few days in all patients. There was no report of any late postoperative hypotony in our study. This rate of hypotony is much lower than that in the study conducted by Pandav et al. where hypotony occurred in 19.4% of the patients and hemorrhagic choroidal detachment occurred in 2 patients. Their surgical technique neither includes the use of mitomycin C nor the ripcord [12].

The removal of the ripcord was needed in 30% of the patients to achieve ideal IOP control. The earliest was done at 3 months postoperatively. It is better to defer the decision of ripcord removal beyond the third month postoperatively to avoid confusion with the transient hypertensive phase.

The results of this study show that use of mitomycin-C during implantation of AADI is a highly effective and relatively safe method of control of IOP in refractory and advanced cases of glaucoma. We had a similar success rate to the similar previous studies on this device with a much lower complication rate which might highlight the importance of the simultaneous use of mitomycin-C and ripcord. The availability and the low cost of the device might give it the edge over other drainage devices in the markets of developing countries with more restricted resources. The current study is limited by the retrospective design, being single-center, and the lack of homogeneity of the included cohort. Further multicenter prospective studies might be needed to assure the efficacy of this relatively new device and to reach the ideal age group candidates and the best surgical technique needed to achieve optimum results.
